# Concurrent chromoblastomycosis and eumycetoma: a unique case of dual neglected tropical fungal diseases in Asia

**DOI:** 10.1371/journal.pntd.0013484

**Published:** 2025-09-02

**Authors:** Wentao Liu, Shuxia Xie, Guoxing Zhu, Shitong Qin, Meirong Li, Songchao Yin, Wei Lai, Chun Lu, Qiaoping Chen, Peiying Feng

**Affiliations:** 1 Department of Dermatology, Third Affiliated Hospital, Sun Yat-sen University, Guangzhou, China; 2 Department of Allergy, Third Affiliated Hospital, Sun Yat-sen University, Guangzhou, China.; Albert Einstein College of Medicine, UNITED STATES OF AMERICA

## Abstract

Chromoblastomycosis (CBM) and mycetoma, as implantation mycoses, have been listed as neglected tropical diseases (NTDs) by the World Health Organization. The concurrent occurrence of these two NTDs in a single patient is extremely rare. A 69-year-old female patient presented with papules on the dorsum of her left hand for over 5 months and nodules on the left lower limb accompanied by ulceration and pain for 20 days. Histopathological examination of the papule on the dorsum of the left hand revealed muriform cells and fungal culture of the tissue identified *Fonsecaea monophora*. Microscopic examination of the purulent secretion from the ulcer on the left lower calf revealed the presence of grains, and the tissue culture result was *Scedosporium apiosperma* complex, with metagenomic next-generation sequencing further identifying *S. dehoogii* as the predominant pathogen. The clinical diagnosis was CBM caused by *F. monophora* combined with eumycetoma due to *S. dehoogii.* The patient was treated with voriconazole at a dosage of 200 mg twice daily for 4 weeks, after which the papules on the dorsum of the left hand and the ulcer on the left lower calf showed gradual improvement. This case represents the first reported instance of concurrent CBM caused by *F. monophora* and eumycetoma due to *S. dehoogii*, providing a novel perspective on the clinical manifestations and early identification of neglected implantation mycoses.

## Author summary

- This study presents the concurrent CBM caused by *Fonsecaea monophora* combined with eumycetoma due to *Scedosporium dehoogii*, which is extremely rare two neglected tropical diseases in a single patient.
- This represents the initial report identifying *S. dehoogii* as a novel causative agent of eumycetoma.
- Both CBM and eumycetoma are curable with antifungal therapy when diagnosed early, as demonstrated in our case report. CBM presented as papules 5 months after trauma, while eumycetoma manifested as abscesses and sinus tracts with purulent discharge containing grains, but not within the tissue, 20 days after trauma.
- Metagenomic next-generation sequencing is not only capable of effectively identifying pathogens in patients with neglected implantation mycoses but also has the potential to assist in the prediction of antifungal susceptibility, as exemplified in our case report.

## Introduction

Neglected tropical diseases (NTDs) represent a diverse group of conditions caused by various pathogens and are associated with devastating health, social, and economic consequences. Chromoblastomycosis (CBM) and eumycetoma are fungal NTDs that cause significant disability and stigma in Latin America, Africa, and Asia [1]. Fungal NTDs typically result from traumatic implantation of fungal elements into the (sub)cutaneous tissues. These infections cannot be easily eradicated because the causative fungi are ubiquitous in the soil, on plant matter, and in the environment. The concurrent occurrence of two NTDs in a single patient is extremely rare. Herein, we report the first case of concurrent CBM caused by *Fonsecaea monophora* and eumycetoma due to *Scedosporium dehoogii* in Asia, providing novel insights into the clinical manifestations and early identification of neglected implantation mycoses.

## Case presentation

**Fig 1:**
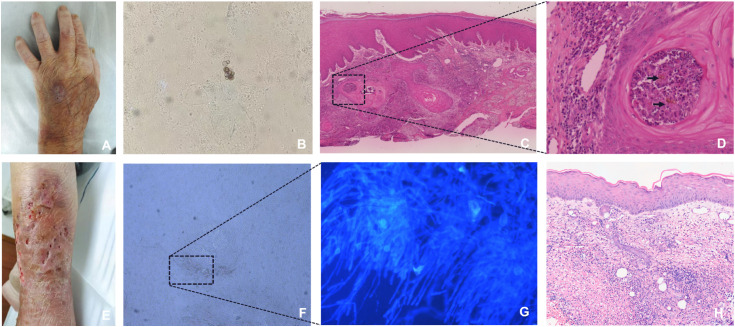
Clinical manifestation of left dorsal hand and left shin skin lesions and etiological and histopathological evidence. **(A)** Papules on the dorsum of the left hand. **(B)** Direct examination of skin scrapings from the lesions showed muriform cells (10%KOH, × 400 magnification). **(C)** The epidermis showed pseudepitheliomatous hyperplasia, and the dermis was infiltrated by mixed cells, which were composed of histiocytes, lymphocytes, multinucleated giant cells, and muriform cells. (HE stain, × 40 magnification). **(D)** Muriform cells in the microabscess (HE stain, × 400 magnification). **(E)** Nodules, abscesses, ulcers, sinus tract, and hyperpigmentation on the lower third of the left calf. **(F)** The grain is white and soft, which is fungal masses formed by interweaving of parallel arranged hyphae, and chlamydospores (10%KOH, × 400 magnification). (G) septate hyphae and chlamydospores (calcofluor white stain, × 400 magnification). **(H)** Mixed inflammatory infiltrate within the dermis, accompanied by focal granulomatous formations consisting of neutrophils and lymphocytes. (HE stain, × 100 magnification).

A 69-year-old female farmer from Heyuan City, a subtropical region in China, was admitted to our hospital with a 5-month history of papular lesions on the dorsum of the left hand following traumatic contact with a chicken coop. Additionally, she presented with a 20-day history of nodular lesions, abscesses, and ulcerations on the left shin, which developed subsequent to a domestic fall. The patient has a 3-year history of rheumatoid arthritis, which has been managed with long-term corticosteroid therapy. On physical examination, three soybean-sized dark red papules with partial coalescence were observed on the dorsum of the left hand, covered by thin brownish crusts (Fig 1A). The lower two-thirds of the left shin exhibited marked hyperpigmentation with scattered mung bean-sized abscesses. These abscesses spontaneously ruptured to form ulcers and sinus tracts draining purulent material, with some ulcers showing confluence (Fig 1E). Laboratory tests for hepatitis B, syphilis, HIV, and T-SPOT were all negative.

## Methods

Microscopic examination of scale specimens obtained from the left-hand papules was performed using potassium hydroxide (KOH) wet mounts. Histopathological analysis of lesional tissue included hematoxylin-eosin staining and evaluation for epidermal and dermal pathology.Fungal culturing of tissue samples from left-hand lesion was conducted on Sabouraud dextrose agar (SDA). Positive cultures were further identified by DNA sequencing to determine the fungal species.

Microscopic assessment of pus from the left shin sinus was performed using KOH mounts and calcofluor staining white. Tissue samples from the left shin sinus underwent SDA culturing and histopathological processing. Lesional tissue from the shin was subjected to metagenomic next-generation sequencing (mNGS) for comprehensive pathogen detection and antifungal susceptibility profiling.

## Results

Microscopic examination of the scale specimen from the left-hand papules revealed muriform cells (Fig 1B). Histopathological examination demonstrated pseudoepitheliomatous hyperplasia of the epidermis and dermal inflammation with various inflammatory cells (Fig 1C), as well as muriform cells within the microabscesses of the dermis (Fig 1D). Fungal culture of tissue and DNA sequence analysis identified the strains isolated from tissue as *F. monophora*.

Microscopic examination of the pus specimen from the left shin sinus revealed a soft white grain (Fig 1F), which was composed of interwoven, parallel hyphae and chlamydospores, visualized under calcofluor white stain (Fig 1G). The culture of the biopsy tissue and pus on the SDA medium resulted in the formation of rapidly growing, cottony-lanose colonies. Histopathological examination showed a mixed inflammatory infiltrate within the dermis, accompanied by focal granulomatous formations consisting of neutrophils and lymphocytes, but no fungal grains were observed (Fig 1H). mNGS analysis of the lesion tissue revealed a total of 402 reads of *S. dehoogii*, covering 93.27% of the total genome, which also indicated that voriconazole exhibits antifungal activity against *Scedosporium* species.

The final diagnosis was concurrent CBM caused by *F. monophora* and eumycetoma due to *S. dehoogii*. The patient was initially treated with intravenous voriconazole at a dosage of 200 mg twice daily. After two weeks of treatment, the lesions on the left lower calf demonstrated significant size reduction. Following discharge from the hospital, the patient was transitioned to oral voriconazole at a dosage of 200 mg twice daily. Follow-up examination revealed that the papules on the dorsum of the left hand had become smooth, with no residual scales. The ulcer on the left lower calf had further healed without any new abscess formation, and mycological examinations were negative.

## Discussion

As tropical and subtropical implantation mycoses, CBM and eumycetoma have different distributions worldwide. CBM prevalence ranges from 1 case per 6,800 in Madagascar to 1 per 86 million in the US [2], while mycetoma is hyperendemic in Sudan and Mauritania at 1.8 to 3.5 per 100,000 [3]. Both infections usually result from cutaneous damage aiding pathogen inoculation. Concurrent infection is rare, with only three verified cases. Earlier cases include a Mexican patient with *Phialophora verrucosa-*associated forearm CBM and *Nocardia brasiliensis*-induced dorsum mycetoma [4]. Following a nine-year progression, an Indian cattle herder was diagnosed with co-localized CBM and eumycetoma on the same leg with muriform cells and grains [5]. Nupur et al. [6] reported a case of dual eumycetoma caused by *Acremonium falciforme* (syn. *Fusarium falciforme*) and *Trematosphaeria grisea* (syn. *Madurella grisea*) in a farmer, presenting with multiple sinuses on the foot and buttock. In contrast, our patient developed CBM due to *F. monophora* and eumycetoma caused by *S. dehoogii* following hand and shin injuries, demonstrating the coexistence of these two distinct fungal infections. As NTDs, CBM and eumycetoma already face limited diagnostic and therapeutic resources. In addition, their co-infection complicated diagnosis, necessitating global attention. Compounding these challenges, their potential for co-infection imposes a dual burden of increased diagnostic complexity and clinical uncertainty, thereby creating a pressing need for global attention.

Lesions of CBM are polymorphous, papular, verrucous, atrophic, tumoral, plaque-like, and mixed, depending on infection duration, host immune status, and geographic location [7]. The initial lesion of CBM is usually solitary, presenting as a small, papular skin lesion. Starting lesions can grow into other skin lesions, creating a polymorphic clinical presentation. Najafzadeh et al. [8] reported a case of CBM caused by *Fonsecaea* in which the skin lesion initially appeared as a small papule, which, over 36 years of development, transformed into a 20 × 30 cm cicatricial erythematous plaque. CBM lesions are categorized as mild, moderate, or severe based on the appearance of the lesions, where mild type is characterized by a single lesion less than 5 cm in diameter. Reported disease durations range from 1 month to 50 years [9]. Notably, epidemiological studies reveal a mean diagnostic delay of 9.2 years, attributable to frequent missed diagnoses during early stages. More importantly, as the disease progresses, lesions may undergo further expansion and become complicated by persistent bacterial coinfection, with rare but critical progression to neoplastic transformation into carcinoma. In the present case, diagnosis was established within 5 months of symptom onset. Clinical examination revealed papular-type lesions and mycological studies confirmed *F. monophora* infection, indicating early-stage detection. To avoid misdiagnosis, we propose fungal infection testing in trauma-related papular skin lesions.

*Scedosporium* is found worldwide in soil, sewage, and decomposing organic matter. This genus has 18 species, five of which are pathogenic and can cause like osteomyelitis, eye infections, mycetoma, sinusitis, and widespread infections [10]. Eumycetoma due to *Scedosporium* is rare, with only 18 instances globally, mostly caused by *S. apiospermum* and one by *S. boydii.* Only 7 of 18 instances had a triad of swollen tissue, sinus tracts, and grain discharge. In 4 cases, no visible grain discharge was observed despite histological identification of grains in biopsies, and 2 cases clinically lacked sinus tract development but showed grains in afflicted tissues. The species *S. dehoogii*, first discovered in 2005, has only been reported in the literature in two cases of subcutaneous hyalohyphomycosis presenting as localized nodular abscesses [11] and one case of fungal osteomyelitis [12]. Our case presents the first confirmed case of eumycetoma associated with *S. dehoogii*. Clinically, the skin lesions were similar to those reported in previous reports of mycetoma caused by *Scedosporium*, which also manifested as abscess, sinus formation, and grains discharge. However, the grains are detected only in pus and not in histopathological sections, which raises a critical question. Is the presence of granules as one of the clinical diagnostic evidence required to be observed simultaneously in both pus and tissue samples? Reviewing previous studies, Lacroix et al. [13] reported a case of mycetoma of the foot caused by *Madurella mycetomatis*, in which clinical examination showed swelling of the right plantar foot with small black grains discharge from the sinus tract, but no grains were found by histologic examination. Conversely, in a chronic case without sinus tracts and grains discharge, a definitive diagnosis was achieved when a biopsy revealed grains embedded in subcutaneous tissue [14]. Therefore, the identification of grains in either pus or histopathological sections can serve adequately as a basis for a diagnosis in our opinion. We speculate that the absence of grains in the tissue is because grains formed in the pus have not yet been encapsulated by the tissue in the early stage of the disease. In addition, secretions and tissue samples should be checked for grains after trauma-induced abscess and sinus tract formation.

mNGS is a high-throughput sequencing technology that can rapidly and comprehensively detect fungal pathogens in samples. mNGS does not rely on traditional culture, and can rapidly identify fungal pathogens and provide drug susceptibility information, reducing the time required for clinical diagnosis and treatment. mNGS identified *S.dehoogii* as the dominant pathogen through sequence proportion in our case, which can identify similar strains and provide a basis for precise diagnosis and treatment. Additionally, mNGS provides antimicrobial susceptibility information which indicates that voriconazole exhibits antifungal activity against both *Scedosporium* and melanized fungi. Eduardo Alvarez et al. [15] also demonstrated the excellent antifungal activity of voriconazole against four strains of *S.dehoogii* isolated from soil in urban areas of Chile. Consequently, this patient received voriconazole treatment and it demonstrated favorable clinical efficacy, further suggesting that in the early stage of treatment, according to the drug susceptibility information provided by mNGS, the empirical use of other antifungal drugs can be avoided so that the patient’s condition can be controlled in time. This case confirmed the dual advantages of mNGS in breaking through the limitations of traditional fungal diagnosis and having the potential to predict drug susceptibility.

This report presents a case of concurrent CBM and eumycetoma. Notably, our study identifies *S.dehoogii* as a novel causative agent of eumycetoma, broadening our understanding of the pathogens involved. We realize that for papular skin lesions or abscess and sinus tract lesions forming, with or without granule discharge, after trauma, mycological testing is essential to avoid missed diagnosis and prevent disease aggravation. Additionally, mNGS shows abilities to diagnose rapidly and accurately and indicate drug susceptibility in this disease, making it an effective tool for diagnosing NTDs.
